# A machine learning algorithm for detecting abnormal patterns in continuous capnography and pulse oximetry monitoring

**DOI:** 10.1007/s10877-024-01155-0

**Published:** 2024-04-15

**Authors:** Feline L. Spijkerboer, Frank J. Overdyk, Albert Dahan

**Affiliations:** 1 Clinical AI Implementation and Research Lab (CAIRELab), Leiden University Medical Center, Leiden, The Netherlands; 2 Trident Health System, South Carolina, North Charleston, United States of America; 3 Department of Anesthesiology, Leiden University Medical Center, Leiden, The Netherlands

**Keywords:** Capnography, Patient safety, Continuous monitoring, Machine learning, Respiratory depression

## Abstract

Continuous capnography monitors patient ventilation but can be susceptible to artifact, resulting in alarm fatigue. Development of smart algorithms may facilitate accurate detection of abnormal ventilation, allowing intervention before patient deterioration. The objective of this analysis was to use machine learning (ML) to classify combined waveforms of continuous capnography and pulse oximetry as normal or abnormal. We used data collected during the observational, prospective PRODIGY trial, in which patients receiving parenteral opioids underwent continuous capnography and pulse oximetry monitoring while on the general care floor [1]. Abnormal ventilation segments in the data stream were reviewed by nine experts and inter-rater agreement was assessed. Abnormal segments were defined as the time series 60s before and 30s after an abnormal pattern was detected. Normal segments (90s continuous monitoring) were randomly sampled and filtered to discard sequences with missing values. Five ML models were trained on extracted features and optimized towards an Fβ score with β = 2. The results show a high inter-rater agreement (> 87%), allowing 7,858 sequences (2,944 abnormal) to be used for model development. Data were divided into 80% training and 20% test sequences. The XGBoost model had the highest Fβ score of 0.94 (with β = 2), showcasing an impressive recall of 0.98 against a precision of 0.83. This study presents a promising advancement in respiratory monitoring, focusing on reducing false alarms and enhancing accuracy of alarm systems. Our algorithm reliably distinguishes normal from abnormal waveforms. More research is needed to define patterns to distinguish abnormal ventilation from artifacts.

**Supplementary Information:**

The online version contains supplementary material available at 10.1007/s10877-024-01155-0.

## Trial Registration

clinicaltrials.gov: NCT02811302, registered June 23, 2016.

**Supplementary Information:**

The online version contains supplementary material available at 10.1007/s10877-024-01155-0.

## Introduction

Opioids are powerful painkillers which are often prescribed after surgery to treat severe pain. However, they come with several adverse effects such as Opioid-Induced Respiratory Depression (OIRD) [2–4]. OIRD can lead to severe and deadly outcomes when not recognized in time, and therefore threatens patient safety [4, 5]. Continuous monitoring allows early detection and intervention of an OIRD and consequently reduces the risk of a fatal outcome [1, 5, 6].

Continuous capnography and pulse oximetry are excellent methods to monitor a patients’ ventilation and oxygenation. However, these monitoring systems are not without their limitations. One major problem with continuous monitoring in non-intubated persons, is the occurrence of artifacts, which falsely trigger the alarm system of the monitor [7, 8]. Estimates suggest that over 70% of alarms may be false, thereby endangering patient safety because clinicians tend to ignore alarms when they are usually false [9–11]. This phenomenon is referred to as alarm fatigue. Therefore, there is a need for an alarm system that is sensitive enough to catch true occurrences of respiratory depression without triggering false alarms.

Continuous capnography and pulse oximetry tracings are affected by many patient-related factors, such as coughing, talking, moving, or equipment-related factors, such as sensor- or calibration errors. Furthermore, there is individual variability in both normal respiratory patterns and the patterns that may indicate respiratory depression. To create an effective alarm system, it is necessary to develop an algorithm that can interpret continuous data, filter out noise, recognize relevant patterns, and make reliable predictions of true events. With the rise of machine learning (ML) and artificial intelligence (AI), the possibilities to accurately analyze capnography data have increased, yet we are still dealing with the challenge of imbalanced datasets, as explained below. True respiratory depression events are infrequent, as they represent irregularities in a generally regular breathing pattern. Normal breathing patterns are much more common than true respiratory depressions. This leads to an inherent class imbalance in the data. This imbalance can bias ML models towards the majority class, resulting in increased misclassifications. Therefore, solving these challenges is key to improving classification of continuous respiratory measurements.

Time series classification (TSC) using a multi-stage approach offers a potential solution to the problem of imbalanced data sets. By initially focusing on the separation of normal breathing and abnormal patterns, we can remedy the class imbalance problem. Following initial classification, more complex models may be applied to the refined ‘abnormal’ dataset in a second stage of the analysis. This sequential method is known to enhance accuracy in anomaly detection and to improve computational efficiency [12, 13]. This makes multi-stage TSC a practical and effective strategy for the classification of continuous capnography and pulse oximetry measurements.

With this approach, the first stage model plays a vital role. The goal of this study is to determine the performance of such a first-stage TSC model. At the same time, the study aims to emphasize the significance and challenge of accurate data labeling when applying ML to respiratory monitoring.

## Methods

### Data and study population

This study entails a secondary analysis on data collected during the observational, prospective PRODIGY trial [1]. After IRB/IEC approval and patient consent, general care floor patients receiving parenteral opioids underwent blinded, continuous capnography and pulse oximetry monitoring with the Capnostream 35 or 20p bedside monitor (Medtronic, Boulder, CO, USA) [1]. The median effective monitoring time was 24 h (IQR 17–26). The data was collected at 16 clinical sites in the United States, Europe and Asia. The included subjects are adults (≥ 18, 20, and 21 years in United States/Europe, Japan, and Singapore, respectively) who were able to wear continuous monitoring equipment. A total of 1,458 patients were included. Details of the PRODIGY study can be found in the article from Khanna et al. (2020) [1].

**Fig. 1:**
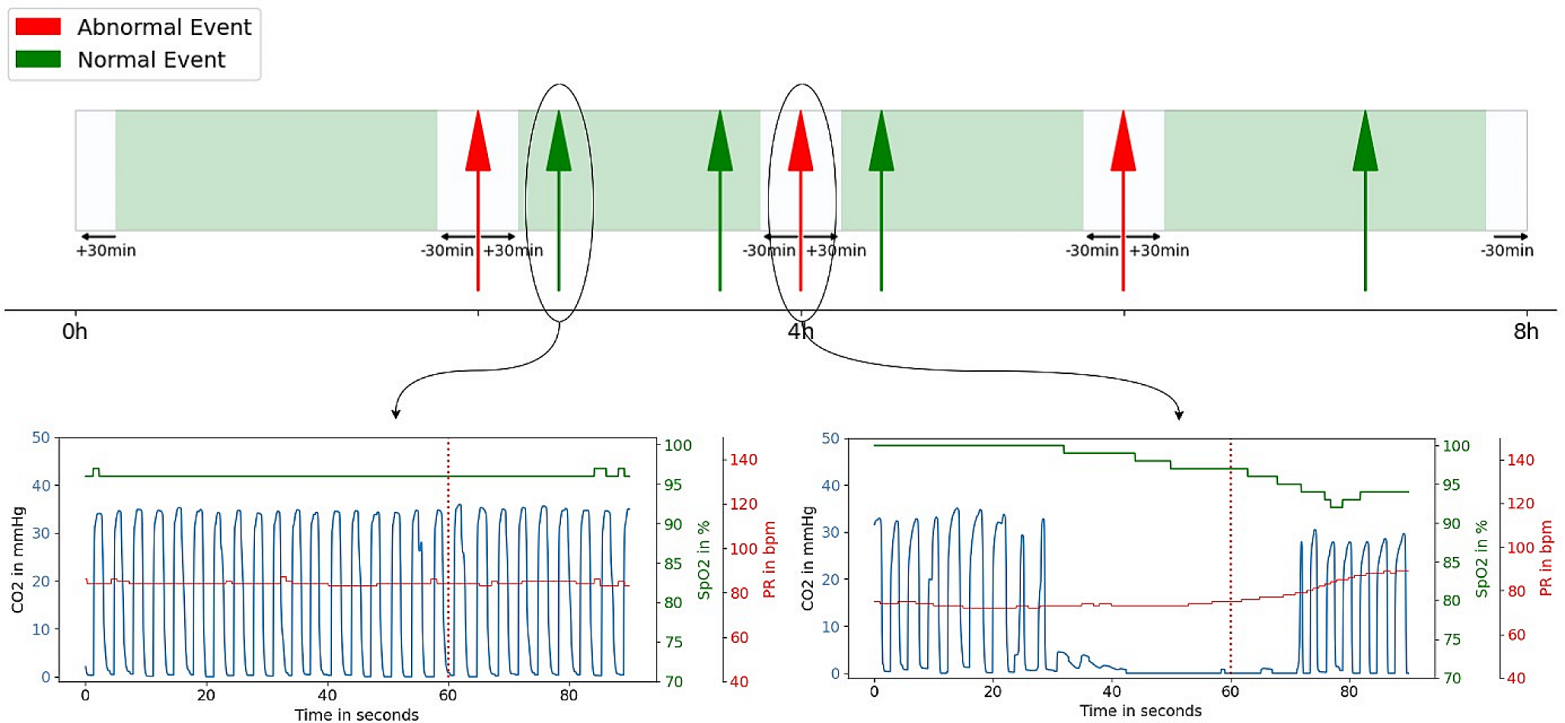
Top panel: Continuous 8-hour recording depicting temporal relationship between abnormal events (red arrows) and normal breathing events (green arrows) Lower panel: Prototypical examples of normal (left) and abnormal (right) segments as seen on capnograph (blue), oximetry (green) and Respiratory Rate (red). These were confirmed by expert panel consensus

Our study utilized 90s segments of combined capnography and pulse oximetry monitoring for each event. An event was defined as the exact timestamp where either abnormal or normal breathing was identified. Abnormal segments started 60s before and ended 30s after the abnormal event. These abnormal patterns were primarily detected automatically via the monitor alarm when certain thresholds limits were breached. The abnormal patterns were then reviewed and confirmed by 9 anesthesiology experts (see section labeling). Normal breathing segments also consisted of 90ss, and were randomly identified from the continuous monitoring tracing, at least 30 min before and 30 min after detecting an abnormal segment. There was no overlap between any of the segments. Figure 1 shows an example of how the events were identified in a continuous measurement of an individual patient. A total of 10,145 segments with a 90s duration were included.

### Labelling and data quality

A team of adjudicators (*n* = 9) was randomly selected from a group of 30 experienced anesthesiologists. All had at least 6 years’ experience in the operating room (OR) and post anesthesia care unit and were knowledgeable with continuous CO_2_ monitoring outside the OR. The labeling task consisted of careful review of the data stream and the adjudication of a label. Each event was assigned one of the following four labels, which consisted of 3 abnormal labels and one normal label:

- Apnea event *(abnormal)*.
- Other Respiratory Depression event *(abnormal)*.
- Artifact *(abnormal)*.
- Normal pattern.

A respiratory depression was defined, as by Khanna et al. as “respiratory rate ≤ 5 breaths/min (bpm), oxygen saturation ≤ 85%, or end-tidal carbon dioxide ≤ 15 or ≥ 60 mm Hg for ≥ 3 min.” [1]. An apnea event was defined as a cessation of breath for > 15s, and an artefact was any segment that showed a prolonged (> 10s) disturbed pattern, and which could not be related to a true respiratory depression. Normal breathing were all segments that showed consistent and regular breathing cycles, where minor deviation with a duration up to 10s, which represented non-significant irregularities in the breathing pattern, were allowed.

Our digital visualization tool served as a platform for data collection, displaying event-specific time sequences, consisting of CO_2_, oxygen saturation (SpO_2_), Pulse Rate (PR) and Respiratory Rate (RR) traces. The tool presented those sequences with a duration of four min before, and two min after an event. The raters could then reassign one of the labels to the presented sequence by selecting one out of the four class labels through input options.

To ensure proper learning and address any uncertainties, raters underwent comprehensive training prior to adjudicating the tracings for the study. This training involved two key components: firstly, the adjudication of 100 tracings, and secondly, an extensive discussion of these tracings in a consensus meeting with the entire team.

Following this learning phase, we conducted the first official round, during which all nine raters independently labeled 300 events. We used the results of this round to analyze if it would be possible to have fewer votes per label, and thereby decrease the workload per rater. We conducted a 5-fold bootstrap analysis to evaluate if the label would change significantly if the input of only seven raters were taken into account, instead of all nine. The Cohen Kappa value of 0.80 (std ± 0.02) indicated that consistency in labels was maintained when votes per event were reduced from nine to seven. As a result, we concluded that having seven votes on a single event would produce a trustworthy label. This allowed us to lessen the burden on raters by reducing the number of events to be labeled per rater. Detailed information on the bootstrap analysis can be found in the in the supplementary information S1.

In the second official round, the nine raters were tasked with revising a total of 3,190 events, where we required seven ratings per event. Consequently, each reviewer was individually responsible for rating 2,485 events, with their individual datasets partially overlapping with those of the other raters. The final label for each event was determined based on the majority vote.

Thus, the nine raters collectively revised a total of 3,490 events, inclusive of all primarily abnormal detections and 168 normal events. These normal segments were selected for revision based on an exploratory visualization of a small subset of the data, which showed deviations from the regular breathing pattern in several segments. It was due to workload considerations that we chose not to revise all 10,145 segments.

The first 300 events were labeled with the consensus of all nine raters, whereas the remaining 3,190 events were revised based on the majority vote of the seven raters involved in assessing each specific event.

### Evaluation of the label revision

Inter-rater agreement was evaluated using two metrics: Fleiss’ Kappa and percent agreement.

The percent agreement for each item was calculated by first determining the label that had the most agreement among raters. Then, the proportion of raters that agreed with this most frequent label was calculated, in relation to the total number of raters. This measure, expressed as a percentage, represents the percent agreement for each item. The overall percent agreement was then obtained by averaging the percent agreement across all items. Fleiss’ Kappa was calculated as described by Fleiss [14]. Both metrics were calculated separately for the two labeling rounds. Furthermore, these metrics were assessed considering the multi-class approach as well as the binary class-definition. The labels resulting from this revision process were then used to create a classification model as described in the next section.

### Development of the classifier

**Fig. 2:**
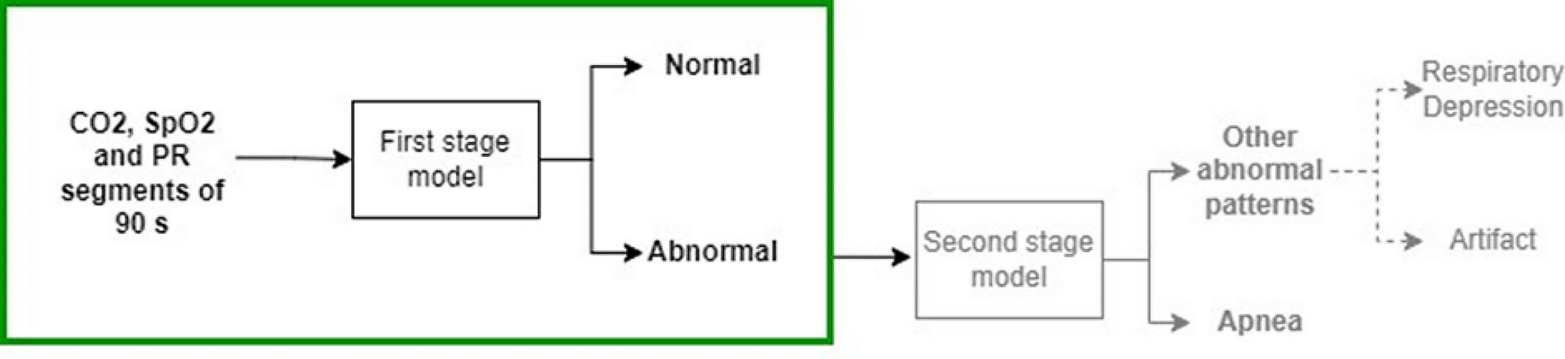
Overview of the multi-stage classifier approach. The current study focusses on the development and evaluation of the first stage classifier, which distinguishes between normal and abnormal segments (green box on the left). Further studies can then focus on the development of a second stage classifier that, e.g., differentiates significant apneas from other abnormal patterns

The goal of this study is to create a first-stage ML classifier to distinguish between normal and abnormal segments of combined capnography and pulse oximetry measurements. This classifier is part of a larger concept which applies multiple sequential classifiers to detect significant respiratory depressions, and which can potentially differentiate artifacts from true respiratory depressions. A general overview of the multi-stage classifier approach is shown in Fig. 2. The rest of this section presents the steps taken in the development and evaluation of the first-stage model.

### Pre-processing

Segments were removed from the dataset when more than 20% of the CO_2_, SpO_2_, or PR measurement was missing or when the CO_2_ value over the entire 90s period was lower than 1.0 mmHg. In cases where less than 20% of the data was missing, a linear interpolation was implemented, followed by a forward and backward fill to address missing values at the beginnings and ends of each segment, respectively.

Using the python packages, TSFresh and NeuroKit2, we extracted 300 relevant features from the raw capnography and pulse oximetry segments [15, 16]. Features with high correlation (> 0.9), low variance (< 0.005), or above 10% missingness were removed, resulting in 208 features for modeling. Subsequently, these features were processed using Scikit-learn’s IterativeImputer for missing data imputation and MinMaxScaler for feature scaling [17].

### Model development and training

We divided our dataset into training and test subsets, maintaining an 80:20 split at the individual subject level. The class ratios in the training and test sets were comparable, with a proportion of 0.63 in the training set and 0.61 in the test set for the negative (normal) class label.

Five ML models were trained, including Gaussian Naive Bayes, eXtreme Gradient Boosting (XGBoost), Random Forest, C-Support Vector (SVC), and K-Nearest Neighbors (KNN). This selection was based on the capability of these models to efficiently handle feature-based classification tasks, while covering a range of different classification methods. These models also have a good balance between computational efficiency, performance and explainability. The classifiers were trained through subject based stratified 5-fold cross validation on the train set.

### Model evaluation

The first-stage model developed in this study is an important first step towards a more precise clinical alarm system for respiratory depression. To assess the performance of the model we used the so-called F_β_ score, which balances precision and recall. Precision, also known as positive predictive value, measures the proportion of true positive identifications among all positive identifications made by the model. A high precision means that the model has a low rate of false positives. On the other hand, recall, also known as sensitivity, measures the proportion of true positives that were correctly identified by the model out of all actual positives. Since it can be fatal to miss a patient with an abnormal breathing pattern, the costs of false negative weights higher than false positives in this first-stage model. Therefore, we chose to train and optimize the models towards an F_β_ score with β = 2. A β value greater than 1, as in our case with β = 2, means that recall is considered more important than precision. In addition, we also assessed further performance measures including accuracy, precision, recall, specificity, AUPRC (Area Under the Precision-Recall Curve) and AUROCc (Area Under the Receiver Operating Characteristic Curve). Note that the second-stage model, which will further classify the abnormal segments as apnea or not, will be aimed towards the reduction of false alarms. Therefore, the second-stage model will be focused more on optimizing the precision score.

Feature importance was calculated with the build in feature importance function of the XGBoostClassifier python package, where importance was based on the number of times a feature appears in a tree.

## Results

**Fig. 3:**
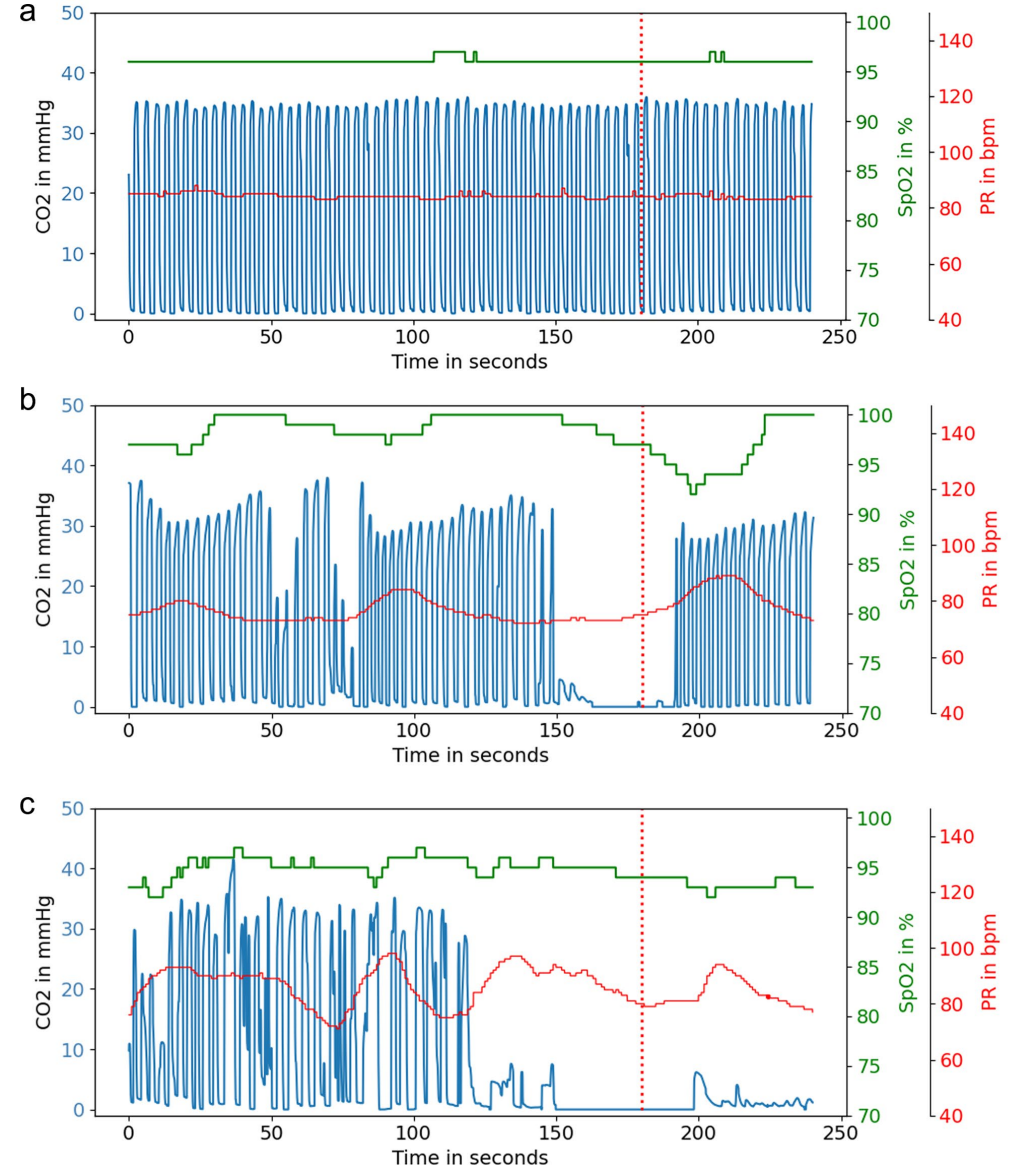
Examples of the combined CO_2_, Oxygen saturation (SpO_2_) and Pule Rate (PR) trace of **a.** a segment of normal breathing, **b**. an apnea episode and **c** a segment which is disturbed by artifacts. The y-axis on the left shows the CO2 concentration in mmHg, and on the right, it shows the SpO_2_ in percentage and the PR in beats per min (bpm). The exact time of each event is defined at the time of the vertical line

Data was derived from 1,458 distinct subjects, with each subject contributing between 1 and 17 segments, and a median of 6 segments per subject. Figure 3 presents examples of combined capnography and pulse oximetry measurements for segments which were labeled as normal, apnea and artifact.

In the effort of refining our dataset, a total of 3490 segments underwent a systematic label revision. Table 1 provides an overview of the number of segments per class label after revision and after preprocessing. It is notable that 2287 segments (22,5% of the total) were discarded during preprocessing step due to missing data, with a significant 2152 (94.1%) of these classified as normal. This shows that a large part of the normal segments contained empty measurements.

**Table 1:** Overview of the number of labeled segments in the revised dataset and in the dataset after preprocessing

	Number of labels in the Revised dataset		Number of labels in the Preprocessed dataset	
**Normal**	7066		4914	
**abnormal**	3079		2944	
Apnea		*406*		*395*
Artifact		*2634*		*2512*
RD		*39*		*37*
**Total**	10,145		7858	

The Inter-rater agreement derived from the label revision is presented in Table 2. It stands out that the Inter-rater agreement for the binary labels is significantly higher than for the multi-class labels. Particularly, the percent agreement with the binary labels yields very satisfactory results, exhibiting levels above 87%. The Fleiss Kappa value was low across all labeling rounds, with the highest value reaching a moderate agreement of 0.48 in the first round of the binary class labels. All other rounds only achieved values less than 0.2, indicating only a slight agreement between raters.

**Table 2:** Inter-rater agreement of the different labeling rounds. The first labeling round included 300 events and 9 votes per event. The second labeling round included 3190 events and 7 votes per event

	Multi-class classification^a^		Binary classification^b^		
	**Fleiss’ kappa**	**Percent agreement**	**Fleiss’ kappa**	**Percent agreement**	
**First round**	0.17	65.6%	0.48	93.6%	
**Second round**	0.01	63.1%	0.03	87.8%	

^a^These measures of Inter-rater agreement are based on multiple class labels. We looked at the agreement on the labels apnea, artifact, RD and normal separately

^b^These measures of Inter-rater agreement are based on binary class labels where we only looked at the agreement based on the normal and abnormal class label

### Model performance

**Fig. 4:**
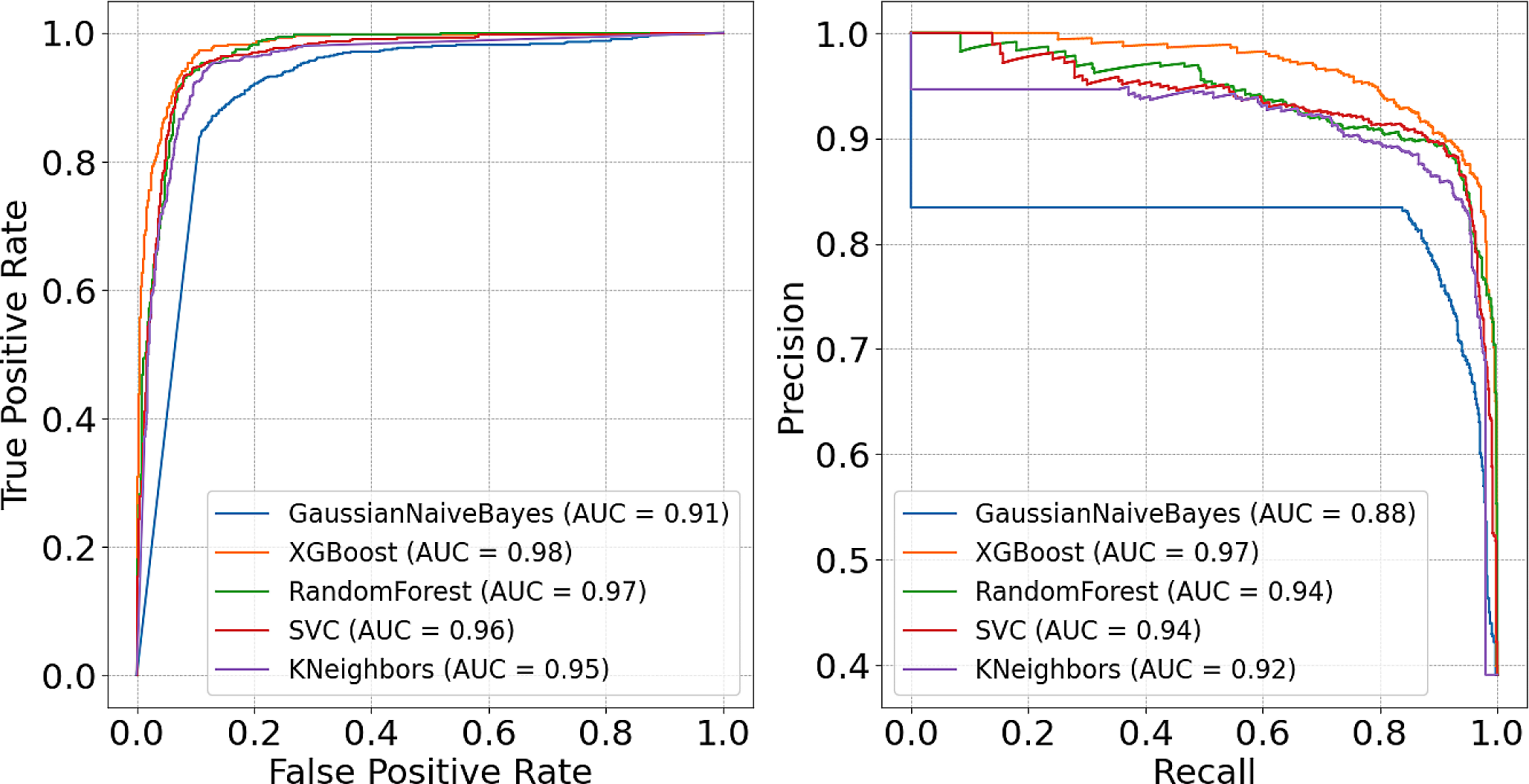
To the left, the Receiver Operating Characteristic (ROC) curve is presented. To the right, the Precision-Recall (PR) curve is shown. The Area Under the Curve (AUC) is presented for each model

The performances of the ML models evaluated on the test set are shown in Table 3. The XGBoost model presented the best capability of correctly classifying abnormal events, based on the F_β_ score with β = 2 of 0.94. This outcome corresponds to the remarkably high recall of 0.98 and a satisfactory precision of 0.83. The XGBoost model also outperformed the other models based on the impressive Area Under the Receiver Operating Characteristics (AUROC) curve score of 0.98 (see Fig. 4). Additionally, when examining the Precision-Recall (PR) curve shown in Fig. 4, the XGBoost model again achieved the highest value, with an Area Under the Precision Recall Curve (AUPRC) of 0.97.

The top 3 important features of our XGBoost model were ‘longest strike below mean CO_2_’^1^, ‘First real Fourier coefficient CO_2_’ and ‘mean SpO_2_’.

**Table 3:** Overview of performance metrics for the test set of the different classification models at a discriminative threshold of 0.5

	F_2_ score	F_1_ score	Accuracy	Precision	Recall	Specificity
GaussianNaiveBayes	0.86	0.84	0.87	0.80	0.88	0.86
XGBoost	**0.94**	0.90	0.91	0.83	**0.98**	0.87
RandomForest	0.93	0.90	0.91	0.85	0.95	0.89
SVC	0.93	0.89	0.91	0.84	0.95	0.89
KNeighbors	0.90	0.89	0.91	**0.86**	0.92	**0.90**

The ‘longest strike below mean (X)’ is a feature defined by the TSFresh python package. It “returns the length of the longest consecutive subsequence in X that is smaller than the mean of X” [15]. When applied to our data, X represents the CO2 trace of our data segment

## Discussion

Our research aimed to develop a classifier to distinguish abnormal from normal breathing patterns in continuous capnography and pulse oximetry monitoring. An important part of this research was the assessment of the data quality, which included the revision of a subset of the data. This process showed a reliable level of agreement among experts, based on the division between normal and abnormal segments. The focus on data quality strengthens the credibility of our findings and forms the basis for the evaluation of our model’s performance.

The best performing classifier was an XGBoost model, which reached a remarkably high recall while maintaining good precision. This demonstrates the model’s capacity for correctly identifying abnormal breathing instances at a relatively low rate of false positive alarms. The design decision to prioritize recall over precision stems from the classifier’s role as a first-stage detector in a multi-stage classification system where any missed abnormal events could be dangerous for the patient. Hence, the model presents a very promising classifier for the initial stage in the multi-stage approach as presented in Fig. 2.

The multi-stage classification approach employed in this study plays a key role in the interpretation of our results. While the presented model can accurately select abnormal patterns, further differentiation of those abnormal patterns is crucial for the clinical utility of the final model. Specifically, future classifiers should be able to distinguish between artifacts or other non-significant events and abnormal respiration, such as apneas, which necessitate an alarm. Despite the dependency on those additional steps, this first-stage model is an important step towards advanced respiratory monitoring, the reduction of false alarms and further insights in patterns of respiratory depression.

Over the last few years, research on automatic capnograph analysis has accelerated. However, no method for the automatic detection of abnormal patterns, which could indicate respiratory depression, has been developed as to our knowledge. Previous research on capnography classification did focus on binary classification of capnography segments, but the classification tasks differ remarkably from ours.

El Badawy et al. developed several models to distinguish between clean and deformed capnograph segments [18]. The most important difference with our study is that they collected their data in a controlled setting, involving 35 healthy subjects, aged between 17 and 33 years, who were seated and monitored for 5 min [18]. Consequently, their dataset does not include any abnormal respiratory patterns and their model will not be able to deal with any kind of capnograph deformity caused by a respiratory depression.

Our study takes a different route by incorporating data sourced from a real clinical setting in a hospital clinical trial, thereby enhancing the real-world applicability of the algorithm. Moreover, our method tolerates short distortions in normal segments, as it is clinically unnecessary to detect all minor anomalies. This emphasis on practical clinical implementation sets our study apart.

Notwithstanding these crucial differences in the data, we share a common objective with the work of El Badawy et al.; we both aim to develop a classifier to differentiate between normal and abnormal capnography segments. The latest model created by this group discusses the delicate balance between specificity and recall, and their most successful model in terms of recall achieved a rate of 94%, against a precision of 80.8% [19]. In comparison, our model improves upon this performance by gaining 4% in recall and 2.2% in precision.

A recent study by Conway et al. presents a classification task very similar to ours, also using the PRODIGY dataset [20]. Their deep learning algorithm classified 15s capnography segments as ‘breath’ or ‘no-breath’ and reached an impressive performance with a recall of 0.96 against a precision of 0.97. Despite many parallels with Conway’s study, our methods were different, making direct comparisons in the performance metrics difficult. The main difference lies in the applied labels. Conway’s study defined a segment as a ‘breath’ when it detected at least one complete respiratory cycle within the 15s interval, with all other segments being classified as ‘no-breath’. Segments were excluded if all 15s samples were zero or any CO_2_ values were missing. This choice of class labels allows that a segment classified as containing ‘breath’ still includes an abnormal breathing pattern, such as hypopnea, and conversely, a ‘no-breath’ segment could easily trigger a false alarm by including an artifact. Furthermore, the exclusion criteria applied may also inadvertently dismiss segments showing significant apnea episodes, which can last for more than 30s. In contrast, our multi-stage approach aims to detect all potentially dangerous respiratory events. Therefore, we focused on capturing all segments with abnormal patterns and thus applied a different classification task.

Another remarkable observation is that much of the existing research in capnography classification relies on analysis of very short time intervals. Often segments only include 15s, which on average captures just three full respiratory cycles, or the input includes only a single breath [18–23]. However, certain abnormal respiratory patterns are more clearly observable over extended periods, such as apnea episodes which can last more than 60s. Our study used segments of 90s which more thoroughly capture these respiratory patterns.

### Limitations

Although the abnormal segments were revised thoroughly, the normal segments were only filtered by a rule-based algorithm to discard any non-valid measurements. This approach may unintentionally have allowed non-normal patterns to be present within the segments labeled as normal. As part of the preprocessing steps, 22.5% of all segments were discarded. The fact that most of these segments were initially labeled as normal indicates a high incidence of noise and empty measurements within the normal segments. However, it also shows the efficiency of the filtering algorithm.

The discrepancy between the high percent agreement and low Fleiss’ kappa in our results is also important to discuss since these values correspond to the so-called ‘Kappa paradox’. In our study, this paradox could be explained by the significant class imbalance present in our dataset. The prevalence of the abnormal class, specifically the artifact class, was exceptionally high within the dataset used for revision, leading to a high chance of agreement, and potentially contributing to the low kappa value. Thus, the kappa value may reflect the prevalence of the majority class more than the actual inter-rater agreement and should be interpreted with care.

The limited number of apnea and respiratory depression class labels might have introduced bias in model training. As most of our abnormal labels are attributed to artifacts, the diversity of abnormal patterns that our model can effectively identify in a real-world setting may be limited. Although our current model is primarily focused on differentiating between normal and abnormal patterns, it is crucial that it accurately identifies clinically significant abnormal patterns. This limited availability of clinically significant abnormal patterns in our dataset may also hinder the development of an accurate second-stage model. Therefore, it is important for future development to enhance the quality and availability of capnography and pulse oximetry segments that showcase various forms of respiratory depression, including apnea episodes.

**Fig. 5:**
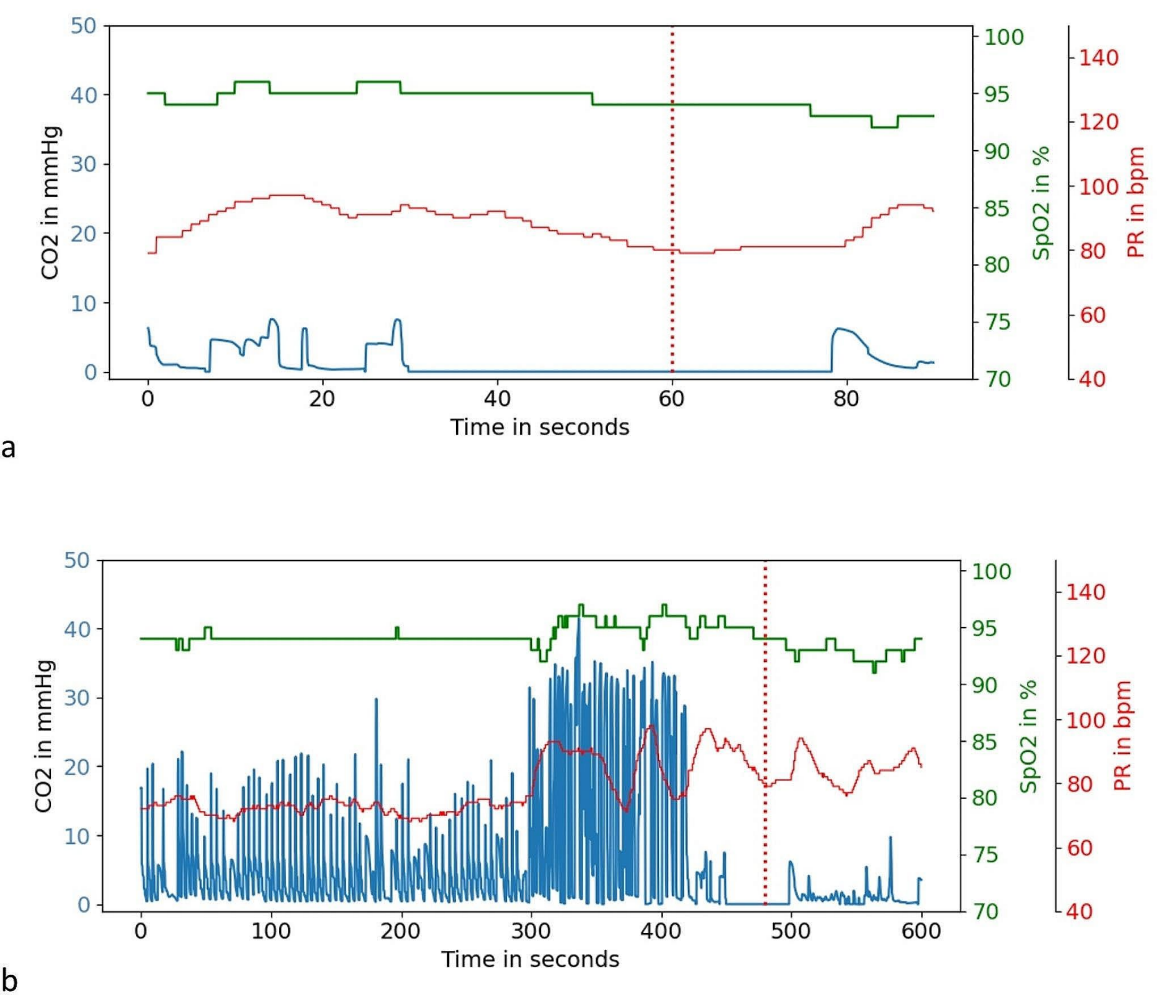
Visualization of the CO_2_, SpO_2_ and Pulse Rate (PR) from the same event, over a duration of (**a**) 90s, and (**b**) 600s

In our methodology, we used 90s segments for our analysis. Although this duration is longer than applied in many similar studies, it is insufficient for capturing all crucial temporal events within the data. A visual comparison of a single measurement but with different durations is provided in Fig. 5. It can be observed that the longer segments are easier to interpret; where the 90s segments leave room for doubt between an artifact or a true apnea episode, the segment based on 600s is highly indicative for movement artifacts. Supporting the value of longer segments, Mieloszyk et al. showed better performance of their capnograph classifier when using a higher number of exhalations as input data [24]. Their final model was based on 80 consecutive exhalations, corresponding on average to a duration of 4–7 min depending on the respiratory rate. Therefore, we recommend subsequent studies to use longer segments as input data.

## Conclusion

Our research builds upon an extensive dataset of continuous capnography and pulse oximetry measurements, sourced from clinical settings across three continents. We focused intensely on using high data quality, as established by expert consensus, to reliably differentiate normal from abnormal patterns. The XGBoost model was found to be the best performing classifier, demonstrating a high recall rate alongside good precision. It effectively identified abnormal breathing instances with a relatively low rate of false alarms. Although our model performs very well as an initial-stage detector in a multi-stage system, the need for subsequent classifiers to further differentiate between abnormal patterns remains essential. Specifically, distinguishing significant respiratory depressions from artifacts is vital. A key challenge in this area is the scarce availability of segments labeled as respiratory depression and apnea. Therefore, we stress the need to concentrate on accurate labeling of significant respiratory events. Overall, this study presents a promising advancement in respiratory monitoring, aiming to minimize false alarms and improve the precision of alarm systems during continuous respiratory monitoring on the ward.

### Electronic supplementary material

Below is the link to the electronic supplementary material.

Supplementary Material 1

## Data Availability

The data that support the findings of this study were made available from Medtronic. Restrictions apply to the availability of these data, which are not publicly available. Data are however available from the corresponding author (Feline L. Spijkerboer, f.l.spijkerboer@lumc.nl) upon reasonable request and with permission of Medtronic.
