# Supplementary material for: A machine learning algorithm for detecting abnormal patterns in continuous capnography and pulse oximetry monitoring
Source: J Clin Monit Comput. 2024 Apr 15;38(4):915–25. doi: 10.1007/s10877-024-01155-0 (PMC11297897; doi:10.1007/s10877-024-01155-0)
Supplement: Supplementary file 1 — Supplementary Material 1 [file 10877_2024_1155_MOESM1_ESM.docx]

Supplementary information

**A Machine Learning Algorithm for Detecting Abnormal Patterns in Continuous Capnography and Pulse Oximetry Monitoring**

Journal of Clinical Monitoring and Computing

Feline L. Spijkerboer, MSc^1^; Frank J. Overdyk, MSEE, MD^2^; Albert Dahan, MD, PhD^3^

^1^Clinical AI Implementation and Research Lab (CAIRELab), Leiden University Medical Center, Leiden, The Netherlands

^2^Trident Health System, North Charleston, South Carolina, United States of America

^3^Department of Anesthesiology, Leiden University Medical Center, Leiden, The Netherlands

**Corresponding Author:** Feline L. Spijkerboer, f.l.spijkerboer@lumc.nl

# **S1** Bootstrap analysis on the number of raters

The revision of labels is a very labor intensive process. With a total of nine raters available, it was necessary to determine whether all raters had to rate all events, or if we could maintain label quality while decreasing the workload per rater. Therefore, this analysis aimed to assess whether utilizing fewer than nine raters per label would significantly impact the final label, which is derived from a majority vote system. A bootstrap analysis was performed on the initial 300 events, each of which has been evaluated by all nine raters during the first round. This analysis explored the effect of limiting the number of votes per label to either five or seven. Table S1 illustrates the methodology used to compute the final label when an event is assessed by only five raters, with the grey fields indicating exclusion from the majority vote in the bootstrap analysis.

Table S1 Example of reducing the number of votes per event from nine to five and its impact on the final label determination. Grey fields indicate the excluded events. The color coding in the last two columns highlights whether the final label remains consistent or changes

| event | label_0 | label_1 | label_2 | label_3 | label_4 | label_5 | label_6 | label_7 | label_8 | Final label 9 | Label bootstrap |
| --- | --- | --- | --- | --- | --- | --- | --- | --- | --- | --- | --- |
| 001_1 | 2 | 2 | 1 | 1 | 2 | 1 | 2 | 1 | 1 | **1** | **2** |
| 001_2 | 3 | 2 | 2 | 3 | 3 | 3 | 2 | 2 | 2 | **2** | **3** |
| 001_3 | 1 | 2 | 1 | 2 | 1 | 1 | 1 | 1 | 1 | 1 | 1 |
| 001_4 | 2 | 3 | 2 | 1 | 1 | 3 | 1 | 2 | 2 | 2 | 1 |
| 001_5 | 2 | 2 | 2 | 2 | 2 | 2 | 2 | 2 | 2 | 2 | 2 |
| 002_1 | 3 | 1 | 2 | 1 | 2 | 3 | 3 | 1 | 2 | 1 | 3 |
| 003_1 | 2 | 2 | 2 | 1 | 2 | 1 | 2 | 1 | 1 | 2 | 2 |
| 003_2 | 2 | 2 | 2 | 2 | 2 | 2 | 2 | 1 | 2 | 2 | 2 |
| 003_3 | 2 | 2 | 2 | 2 | 2 | 2 | 2 | 1 | 2 | 2 | 2 |
| 003_4 | 2 | 2 | 2 | 2 | 2 | 2 | 2 | 1 | 2 | 2 | 2 |
| 005_1 | 4 | 2 | 2 | 2 | 3 | 3 | 3 | 3 | 3 | 3 | 2 |

Since the label and level of agreement might vary depending on the specific raters selected per event, it was deemed fair to apply bootstrapping multiple times across different subsets and assess the average outcome. Therefore, the bootstrapping process was performed five times, where each iteration employed a different interval for the raters to be left out. Subsequently, the percent agreement and Cohen Kappa were calculated for each iteration and then averaged. The results of this analysis are shown in Table S2.

Table S2 Results of the bootstrap analysis. The percent agreement and Cohen’s Kappa values for each iteration is shown based on the majority vote for seven raters per event. It also presents the mean and standard deviation of these five rounds. Additionally, the mean and standard deviation based on five raters per event is shown.

|  | Trial 1 | Trial 2 | Trial 3 | Trial 4 | Trial 5 | Based on 7 raters per event  (mean +- std) | *Based on 5 raters per event*  *(mean +- std)* |
| --- | --- | --- | --- | --- | --- | --- | --- |
| **Percent agreement** | 91.3% | 90.0% | 92.3% | 90.0% | 90.3% | ***90.8% (±0.9)*** | ***83.0% (±2.1)*** |
| **Cohen Kappa** | 0.81 | 0.78 | 0.83 | *0.78* | 0.79 | ***0.8 (±0.02)*** | ***0.65 (±0.05)*** |

The results show a Cohen’s Kappa of 0.8 and a percent agreement of 90.8%, indicating a high level of consistency in the final label assignment when the number of votes per event is reduced from nine to seven. It can also be observed that the percent agreement for the five-vote scenario was 83.0%, with a Cohen's Kappa of 0.65, represent a substantial decrease in consistency. Given these outcomes, it was determined that using five raters per event would compromise the robustness and reliability of the labels. Therefore, we decided that the seven-vote approach was a good balance between the reduction of the workload while maintaining label quality. The second round of the label revision was thus based on seven raters per event.
